# Supplementary material for: Commensal Escherichia coli Strains of Bovine Origin Competitively Mitigated Escherichia coli O157:H7 in a Gnotobiotic Murine Intestinal Colonization Model with or without Physiological Stress
Source: Animals (Basel). 2023 Aug 10;13(16):2577. doi: 10.3390/ani13162577 (PMC10451813; doi:10.3390/ani13162577)
Supplement: Supplementary file 1 [file animals-13-02577-s001.zip › animals-2506093-supplementary.pdf]

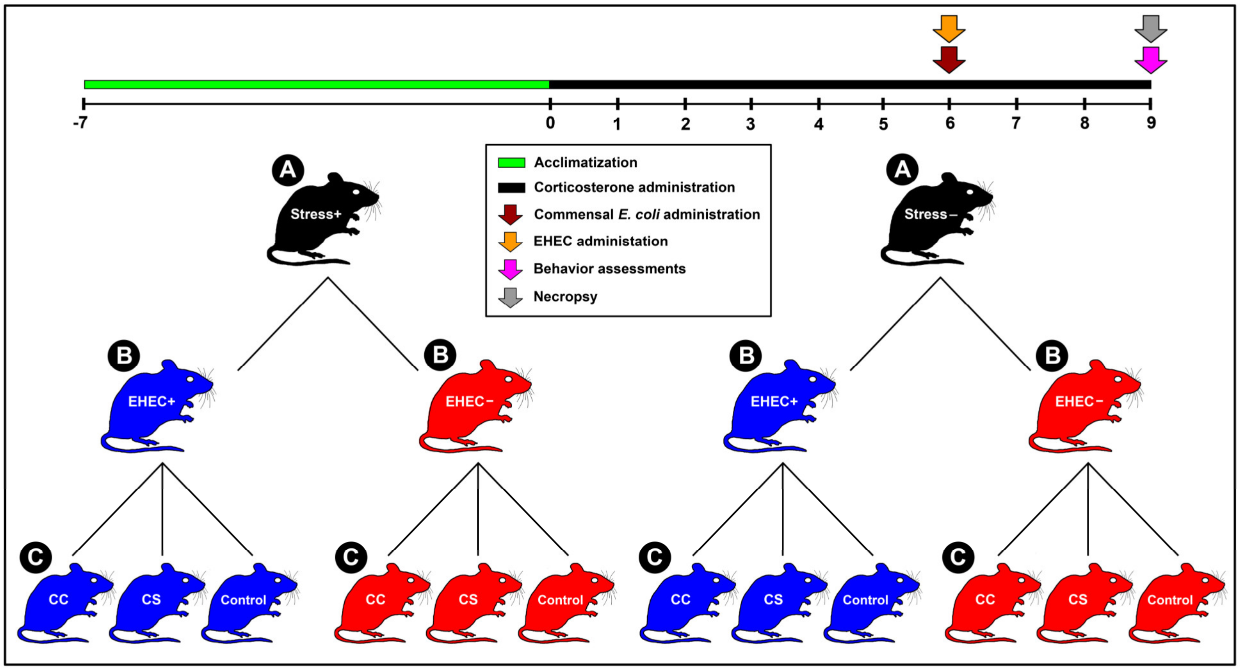

Figure S1. Schematic design for experiment two. The experiment was arranged as a two (**A**; ± stress) by two (**B**; ± enterohemorrhagic *Escherichia coli* O157:H7 [EHEC] FRIK-2001) by three (**C**; commensal *E. coli* treatments) factorial design arranged as a completely randomized design with three replicate germ-free or gnotobiotic mice per treatment combination (36 mice total). Commensal *E. coli* strains were cultured communally (CC) or separately (CS), whereas the Control treatment mice were orally administered phosphate buffered saline alone. Stress was incited by the administration of corticosterone via drinking water, and EHEC and commensal *E. coli* strains were administered orally to mice via consumption of Jell-O formulated with the bacteria. See the manuscript text for additional information.

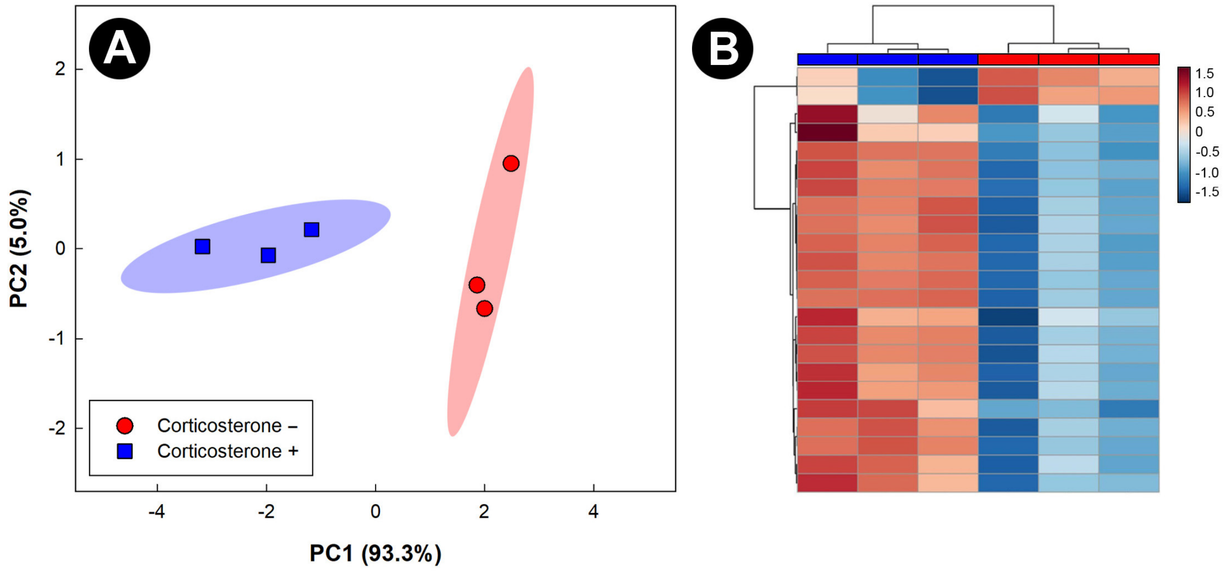

Figure S2. Principal component analysis (PCA) score plot and heat map of metabolite bins in livers of mice administered corticosterone in water for 9 days (corticosterone +; blue markers and bars) or mice administered water free of corticosterone (corticosterone –; red markers and bars). (**A**) PCA score plot, where each marker represents one mouse, and the shaded ellipses represent 95% confidence intervals. The x- and y-axes show principal components one and two, respectively, with the number in brackets indicating the percent variance of each component. (**B**) Heat map showing the degree to which metabolite bins were up- or down-regulated (i.e., denoted with red and blue coloration, respectively). The dendrogram associated with the heat map illustrates the results of the unsupervised hierarchical clustering analysis.

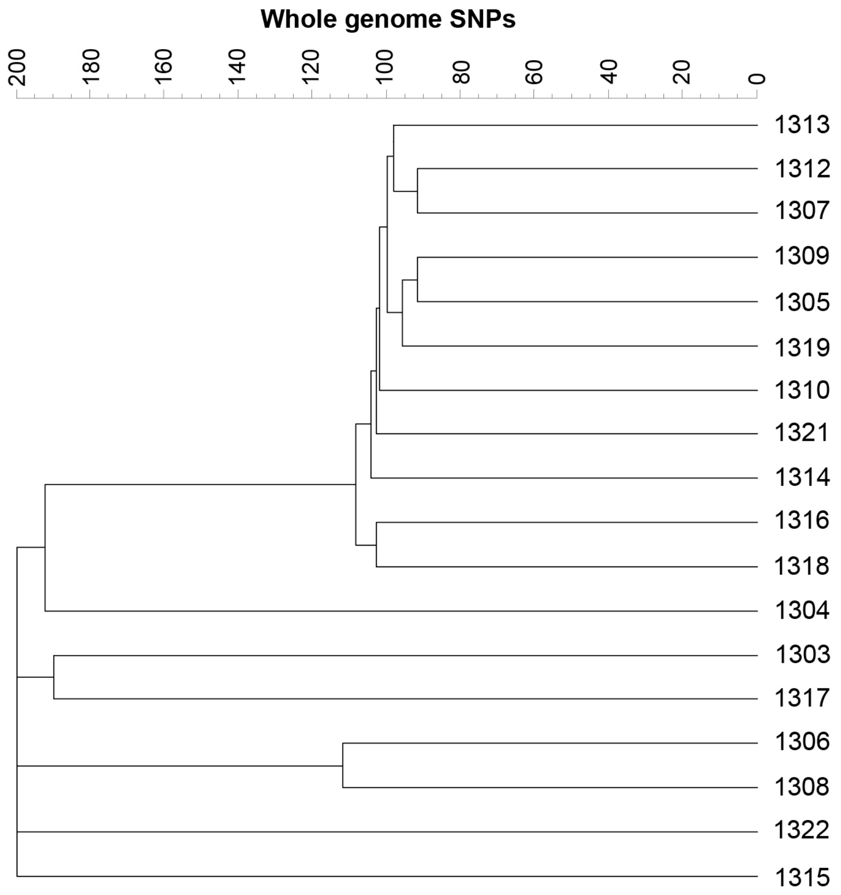

Figure S3. Single nucleotide polymorphism dendrogram showing phylogenetic differences among the 18 commensal *Escherichia coli* isolates that were evaluated in gnotobiotic mice.

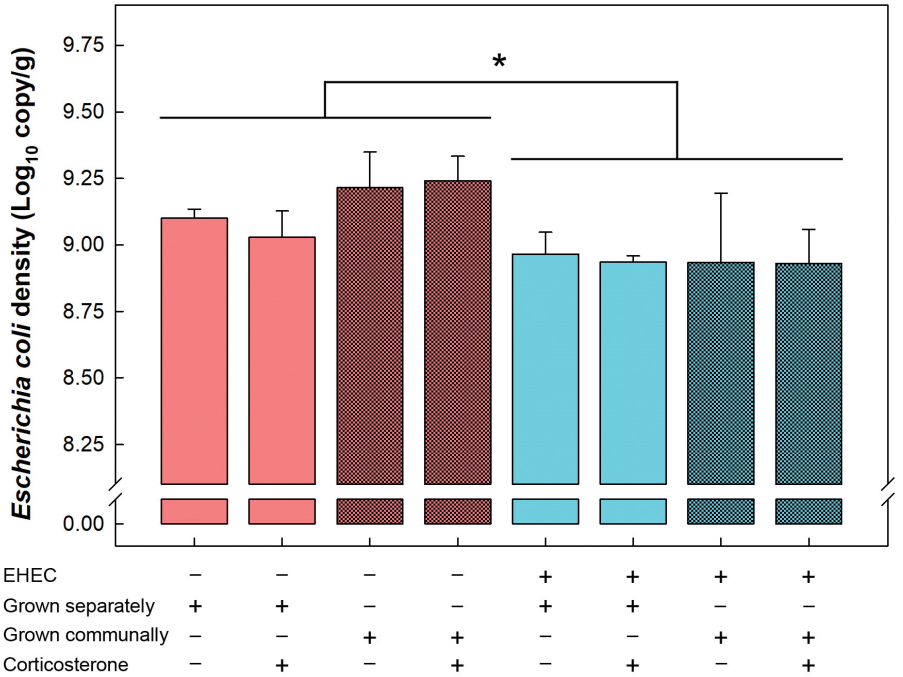

Figure S4. Densities of total commensal *Escherichia coli* cells in distal colonic digesta. Treatments are ± enterohemorrhagic *E. coli* O157:H7 (EHEC), ± commensal *E. coli* strains grown communally or separately, and ± corticosterone administered to mice. Densities were determined using quantitative PCR with primers specific for total bacteria (i.e., all commensal *E. coli* strains as well as the EHEC strain, FRIK-2001). Histogram bars indicated with as asterisk differ ($p = 0.036$) (i.e., densities of commensal *E. coli* cells were reduced in mice that were administered EHEC O157:H7 (i.e., FRIK-2001).

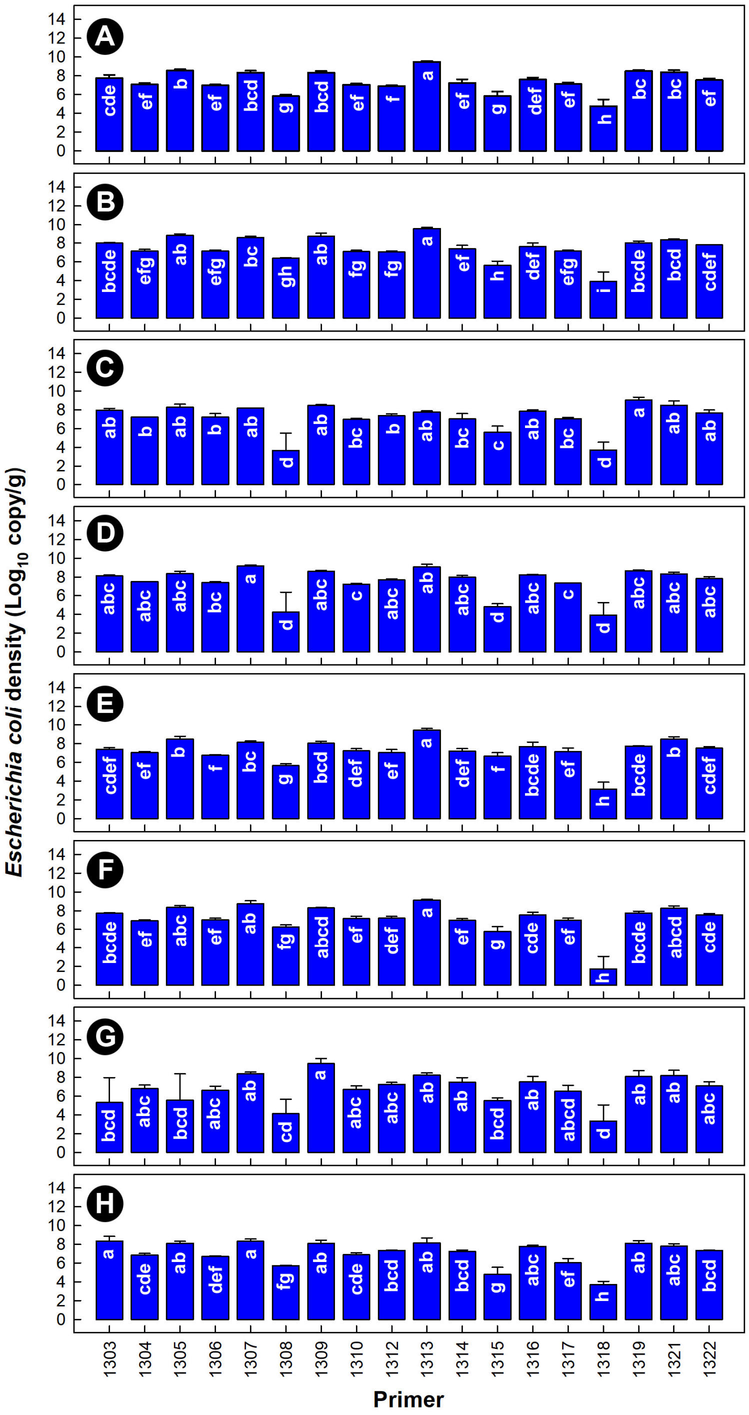

Figure S5. Densities of individual commensal *Escherichia coli* strains in distal colonic digesta. (**A**) enterohemorrhagic *Escherichia coli* O157:H7 (EHEC) (–), Cultured Separately (CS), corticosterone (–). (**B**) EHEC (–), CS, corticosterone (+). (**C**) EHEC (–), Cultured Communally (CC), corticosterone (–). (**D**) EHEC (–), CC, corticosterone (+). (**E**) EHEC (+), CS, corticosterone (–). (**F**) EHEC (+), CS, corticosterone (+). (**G**) EHEC (+), CC, corticosterone (–). (**H**) EHEC (+), CC, corticosterone (+). Densities were determined using quantitative PCR with designed primers that were specific for individual commensal *E. coli* strains. Within individual graphs, histogram bars not indicated with the same letter differ ($p \leq 0.050$).

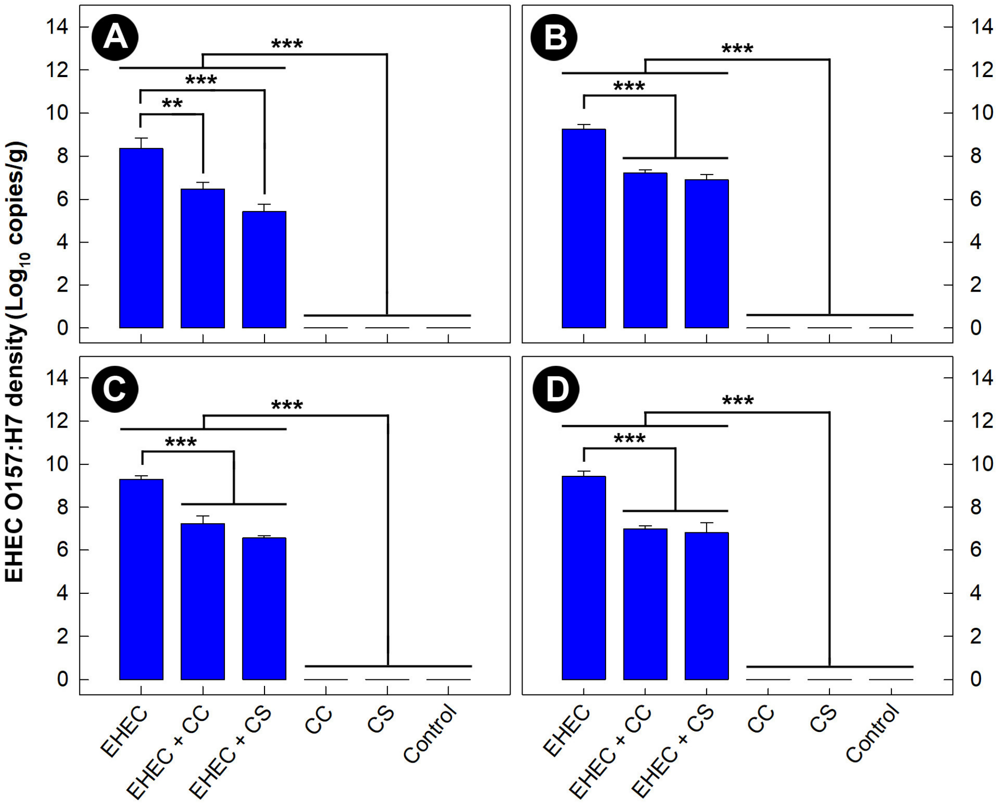

Figure S6. Densities of enterohemorrhagic *Escherichia coli* O157:H7 (EHEC) in intestinal digesta. (**A**) Ileum. (**B**) Cecum. (**C**) Proximal colon. (**D**) Distal colon. Treatments include mice that were inoculated with EHEC (i.e., EHEC, EHEC + CC, EHEC + CS) or with phosphate buffer alone (i.e., CC, CS, Control). Commensal *E. coli* strains administered to mice were grown communally (i.e., CC) or separately (i.e., CS). EHEC and Control treatment mice were not administered commensal *E. coli*. EHEC densities were determined using quantitative PCR with primers that were specific for FRIK-2001. Histogram bars indicated with asterisks differ (** $p < 0.010$; and *** $p < 0.001$). As there was no impact of corticosterone on densities of EHEC, data were averaged over the corticosterone treatment.

Table S1. Primers and annealing temperatures used to quantify enterohemorrhagic *Escherichia coli* O157:H7 (EHEC) strains. See the manuscript text for PCR-specific information.

Strain	Forward primer	Reverse primer	Annealing temperature (°C)
EDL933	TGACCGCTTACGCAGTTCGC	AATGGTTGCTGCCACGGCTC	68
FRIK-2001	GGATGACGGTGCCTGTGCTG	GGCTGGCGGGAGCTACCTAA	68
TW14359	ATCGGTGGCTGGAATGGGCT	AAGTGCAACTGGTGCGTGCT	68
ECI-1375	CGCTCGCCCTAAGATGGGGA	TTCGACGGCTTTCGCTGACG	68
ECI-1911	CACACTGTCCGGTGATGCCG	CGCGCTGTTCAGCATGAGGT	68

Table S2. Primers and annealing temperatures used to quantify commensal *Escherichia coli* strains originally isolated from cattle. See the manuscript text for PCR-specific information.

Strain	Forward primer	Reverse primer	Annealing temperature (°C)
1303	CATATAGTGGTGAACCCGATCC	ACGCCCTAAAGCAGTGAAA	60
1304	CGGACGGTCTATAACCTTGAAC	TTGTGTTGCCGCTGTACT	58
1305	CACCTGACCATCTCTCGTTTAT	GCGAATTGAGGCTTATGGTTTAG	60
1306	GCTCTTCTGCAACGGTTTATG	CGATGTGCCATATGGGTTTAATG	59
1307	CATTTCAGATGTGCGGGAAAG	GCCGTACCTCTAAGTTTCTGAG	60
1308	ATCTCATGTCAGACAAGGAGTTTAT	GAGACCAGACAGGTTTAGGTATTT	58
1309	ACCAGCCTTTCCAGTAAGTTC	CCACATCCCGTCACACAATA	60
1310	GCTGGGAGGATTCCCAATATC	AAGTGTTCGTTGTGGTGATAGA	58
1312	GACACGAACGGATGAAGGAA	TCGTTGTAACCATCGCCATAA	58
1313	CCGGTAATGGCGGTTGTAAT	GGAACAGACGATGCAGGTATTT	60
1314	GGCTGACGTACTGGATCATTTA	ACACTCGCTGAGGCTTTC	60
1315	GATGGTGACAGCCTTGTACTT	ACTTCTCGGCGACTGAAATG	58
1316	CCACTCACTTTGTCCTGGTT	GCTCTGAGGCGTTTGGTATTA	60
1317	CGTGCACAGATGGTTGATAGA	CCGACGAACTGATGATGTAACT	58
1318	TTCACCCGGGAATATCCTTTG	CCGCGATTACCTTGGTTACTAT	58
1319	GGTTTCGGGCTTACTTGTATCT	CGTCTAATGGAGGCATGGAAA	58
1321	GATGATGTATCTAGGCAGGGTTTAT	CAGTCTCTCTTCAAAGCATCCA	60
1322	CCATGAATTACGTTTCTCAGCTAAT	AAGGACGTAAGGCTCGTAGA	58

Table S3. Health assessment scoring system.

Category	Score	Definition
General activity	0	Bright and alert
	1	Slow to move, stays in nest area, restless/agitated or separated
	2	Reluctant to move, not bright or alert
	3	Reluctant to move even if prodded gently and depressed, hunched, eyes partly closed
	4	Moribund
Hair coat appearance and grooming behavior	0	Fur coat shiny and smooth, regular grooming behavior
	1	Reduced grooming behavior, fur coat dry, beginning to huddle
	2	Huddled, fur coat rough, dry, and stands up
	3	Huddled, ungroomed, severe piloerection observed
Vocalization	0	No vocalization
	1	Occurrence of vocalization

Table S4. Predicted commensal *Escherichia coli* strain phenomics (subtype designations).

	Conventional MLST									Phylotype		Serotype prediction		
**Strain**	**adk**	**fumC**	**gyrB**	**icd**	**mdh**	**purA**	**recA**	**ST**	**Clonal complex**	**Profile**	**Clermont phylotype**	**O-Type**	**H-Type**	**Serotype**
1303	6	445	326	12	282	178	247	3381		TspE4: –; arpA: +; chu: +; yjaA: +	E	O11	H25	O11:H25
1304	8	11	4	8	8	9	2	1495		TspE4: –; arpA: +; chu: -; yjaA: +	A	O133	H4	O133:H4
1305	6	4	32	16	12	8	7	164		TspE4: +; arpA: +; chu: -; yjaA: –	B1	O45	H42	O45:H42
1306	34	36	39	87	67	16	4	349	ST349	TspE4: +; arpA: +; chu: +; yjaA: –	D	O166	H15	O166:H15
1307	119	150	12	16	24	8	6	661		TspE4: +; arpA: +; chu: –; yjaA: –	B1	O174	H2	O174:H2
1308	34	36	28	25	28	16	4	70		TspE4: –; arpA: +; chu: +; yjaA: –	D	O23	H15	O23:H15
1309	43	41	15	18	11	7	6	101	ST101	TspE4: +; arpA: +; chu: –; yjaA: –	B1	O21	H21	O21:H21
1310	6	4	4	18	24	5	14	56	ST155	TspE4: +; arpA: +; chu: -; yjaA: –	B1	O25	H16	O25:H16
1312	6	19	5	1	335	8	7	—		TspE4: +; arpA: +; chu: -; yjaA: –	B1	–	H2	–:H2
1313	6	19	3	16	11	8	6	1727		TspE4: +; arpA: +; chu: -; yjaA: –	B1	O83	H7	O83:H7
1314	6	95	4	18	11	7	14	1304		TspE4: +; arpA: +; chu: -; yjaA: –	B1	O163	H7	O163:H7
1315	76	195	19	13	17	28	467	6527		TspE4: +; arpA: –; chu: +; yjaA: +	B2	O2	H10	O2:H10
1316	6	23	4	18	7	7	6	1001		TspE4: +; arpA: +; chu: -; yjaA: –	B1	O8	H2	O8:H2
1317	83	12	155	30	1	1	17	1629		TspE4: –; arpA: +; chu: +; yjaA: –	E	–	H31	–:H31
1318	9	4	33	18	11	8	6	336	ST278	TspE4: +; arpA: +; chu: -; yjaA: –	B1	O151/O118	H27	O151/O118:H27
1319	9	4	5	88	9	8	7	8620		TspE4: +; arpA: +; chu: -; yjaA: –	B1	O8	H16	O8:H16
1321	6	19	14	16	11	12	2	1079		TspE4: +; arpA: +; chu: -; yjaA: –	B1	O6	H49	O6:H49
1322	46	26	208	6	4	16	4	1567		TspE4: –; arpA: +; chu: +; yjaA: –	D	–	H48	–:H48

Table S5. Predicted commensal *Escherichia coli* strain phenomics (antimicrobial resistance).

	Antimicrobial resistance prediction		
**Strain**	**Genotype**	**Resistance**	**Plasmid**
1303	None	Sensitive	None
1304	None	Sensitive	None
1305	None	Sensitive	IncFIA, IncFIB(AP001918)
1306	None	Sensitive	None
1307	None	Sensitive	None
1308	None	Sensitive	IncFIA
1309	aph(3'')-Ib, aph(6)-Id, sul2, tet(B)	StrR, KanR, SulR, TetR[a]	IncFIB(pB171)
1310	aph(3'')-Ib, aph(6)-Id, sul2, tet(B)	StrR, KanR, SulR, TetR[a]	None
1312	None	Sensitive	IncX1
1313	None	Sensitive	None
1314	None	Sensitive	IncFIA
1315	None	Sensitive	IncFIB(AP001918), IncX1
1316	None	Sensitive	IncFIB(pB171)
1317	None	Sensitive	IncFIB(AP001918), IncI2(Delta)
1318	None	Sensitive	IncFIB(AP001918)
1319	None	Sensitive	None
1321	None	Sensitive	IncFIA, IncFIB(AP001918)
1322	None	Sensitive	IncFIA

[a]Str, streptomycin; Kan, kanamycin; Sul, sulfisoxazole; Tet, tetracycline

Table S6. Predicted commensal *Escherichia coli* strain phenomics (virulence factors).

Strain	Predicted virulence factors[a]
1303	*chuA; eilA; espY2:000868321; fdeC; fimH; hlyE; nlpI; ompT; terC; terC; traJ; traT; yehA; yehB; yehC; yehD*
1304	*capU; csgA; fimH; fimH; hlyE; iss; nlpI; sitA; terC; terC; yehA; yehB; yehC; yehD*
1305	*csgA; fdeC; fimH; hlyE; iss; lpfA; nlpI; ompT; ompT; terC; terC; traJ; traT; yehA; yehB; yehC; yehD*
1306	*aslA; chuA; eilA; fimH; hlyE; iss; kpsE; kpsMII_K5; nlpI; shiB; terC; terC; yehB; yehC; yehD*
1307	*csgA; fdeC; fimH; hlyE; iss; lpfA; lpfA; nlpI; terC; terC; yehA; yehB; yehC; yehD*
1308	*aslA; astA; chuA; eilA; fimH; gad; gad; hlyE; kpsE; kpsMII; kpsMII; nlpI; papC; shiB; terC; terC; tia; traJ; traT; yehA; yehB; yehC; yehD*
1309	*F17A; F17C; F17D; F17G; afaA; afaB; afaB; csgA; fdeC; fimH; hlyE; iss; lpfA; nlpI; ompT; ompT; shiB; terC; terC; tia; traT; yehA; yehB; yehC; yehD*
1310	*F17A; F17C; F17D; F17G; csgA; fdeC; fimH; fyuA; gad; hlyE; irp2; iss; lpfA; nlpI; ompT; terC; terC; tia; traT; yehA; yehB; yehC; yehD*
1312	*csgA; fdeC; fimH; gad; hlyE; lpfA; nlpI; terC; terC; yehA; yehB; yehC; yehD*
1313	*fimH; gad; hlyE; iss; lpfA; nlpI; ompT; shiB; terC; terC; yehA; yehC; yehD*
1314	*csgA; fdeC; fimH; gad; hlyE; iss; lpfA; nlpI; ompT; terC; terC; traT; yehA; yehB; yehC; yehD*
1315	*aslA; cba; cdt-VB; chuA; cib; clbB; cma; cnf2; etsC; fimH; focC; focG; fyuA; gad; hlyA; hlyF; iroN; irp2; iss; kpsE; kpsMII_K1; mchB; mchC; mchF; mcmA neuC; nlpI; ompT; sfaD; sitA; terC; terC; traT; usp; vat; yehA; yehB; yehC; yehD; yfcV*
1316	*csgA; fdeC; fimH; hlyE; iss; lpfA; nlpI; ompT; terC; terC; traJ; traT; yehA; yehB; yehC; yehD*
1317	*air; chuA; eilA; eltIIAB-c2; espY2:000868321; fimH; hlyE; nlpI; ompT; terC; terC; traT; usp; yehB; yehC; yehD*
1318	*F17A; F17C; F17D; F17G; cdt-IIIB; cnf2; csgA; fdeC; fimH; hlyE; hra; iss; iucC; iutA; nlpI; ompT; ompT; terC; terC; traT; yehA; yehB; yehC; yehD*
1319	*csgA; fdeC; fimH; hlyE; iss; lpfA; nlpI; ompT; tcpC; terC; terC; yehA; yehB; yehC; yehD*
1321	*anr; astA; csgA; fdeC; fimH; hlyE; iss; lpfA; nlpI; ompT; ompT; shiB; terC; terC; traJ; traJ; traT; traT; yehA; yehB; yehC; yehD*
1322	*aslA; chuA; eilA; fimH; gad; hlyE; nlpI; ompT; terC; terC; traJ; traT; yehA; yehB; yehC; yehD*

[a]See Joensen et al. [1] and Malberg Tetzschner et al. [2] for specific information on the virulence factors presented herein.

Table S7. Frequency of commensal *Escherichia coli* strains recovered from the propagation culture medium (i.e., for strains propagated communally).

Strain	Frequency (%)[a]
1303	2.2
1304	7.7
1305	8.8
1306	1.1
1307	2.2
1308	1.1
1309	5.5
1310	2.2
1312	13.2
1313	1.1
1314	9.9
1315	3.3
1316	5.5
1317	4.4
1318	1.3
1319	9.9
1321	9.9
1322	4.4

[a]The total number of colonies examined was 300.

References

1. Joensen, K.G.; Scheutz, F.; Lund, O.; Hasman, H.; Kaas, R.S.; Nielsen, E.M.; Aarestrup, F.M. Real-time whole-genome sequencing for routine typing, surveillance, and outbreak detection of verotoxigenic *Escherichia coli*. *J Clin Microbiol* **2014**, *52*, 1501-1510, doi:10.1128/JCM.03617-13.
2. Malberg Tetzschner, A.M.; Johnson, J.R.; Johnston, B.D.; Lund, O.; Scheutz, F. In silico genotyping of *Escherichia coli* isolates for extraintestinal virulence genes by use of whole-genome sequencing data. *J Clin Microbiol* **2020**, *58*, doi:10.1128/jcm.01269-20.
